# Maltose promotes crucian carp survival against *Aeromonas sobrial* infection at high temperature

**DOI:** 10.1080/21505594.2020.1787604

**Published:** 2020-07-22

**Authors:** Ming Jiang, Li-Fen Yang, Jun Zheng, Zhuang-Gui Chen, Bo Peng

**Affiliations:** a The Third Affiliated Hospital, State Key Laboratory of Biocontrol, Guangdong Key Laboratory of Pharmaceutical Functional Genes, School of Life Sciences, Sun Yat-sen University, University City, Guangzhou, China; b Laboratory for Marine Biology and Biotechnology, Qingdao National Laboratory for Marine Science and Technology, Qingdao, China; c Southern Marine Science and Engineering Guangdong Laboratory (Zhuhai), Zhuhai, China; d Faculty of Health Sciences, University of Macau, Macau SAR, China

**Keywords:** Metabolomics, maltose, *Aeromonas sobrial*, crucian carp, immune response

## Abstract

Temperature influences fish’s susceptibility to infectious disease through an immune response. However, the mechanism underlying this regulation is yet to be elucidated. In this study, we compared the susceptibility of crucian carp that were grown at 18°C and 33°C, respectively, to *Aeromonas sobrial* infection and found that crucian carp was more susceptible when grown at 33°C. These distinct susceptibilities of fish at different temperatures to infection may partially be explained by their differences in the metabolism as revealed by comparative metabolomics profiling: crucian carp demonstrated enhanced TCA cycle but reduced fatty acid biosynthesis; Our study also found that maltose was the most suppressed metabolite in fish grown at 33°C. Importantly, exogenous injection of maltose enhances crucian carp survival grown at 33°C by 30%. Further study showed that exogenous maltose downregulated the production of several cytokines but enhanced the lysozyme (*lyz*) and complement component *c3*, which involves the humoral innate immunity. Our results suggest that maltose promotes the survival of crucian carp likely through fine tuning the immune gene expression, and this finding provides a novel approach to manage bacterial infection.

## Introduction

The fishery is recognized as an important sector in supplying nutrients to tackle micronutrient deficiency that causes one million premature deaths annually [1,2]. Due to the decline in wild fish stocks, aquaculture has become a replacing strategy to sustain the fish stocks worldwide. As in the form of intensive farming, infectious disease is a major threat to the production in aquaculture [3]. Environmental factors, such as oxygen, water temperature, pH, and ammonia, influence the immune system of fish [4–7]. Temperature is an important factor among these, and the outbreak of infectious diseases in aquaculture was frequently observed in the spring and fall when the water temperature is between 22°C and 30°C [8]. It has been showed that immune response to vaccination and phagocytosis was negatively regulated by extreme temperature [9]. However, the mechanism of how temperature regulates the immune response of the fish is still poorly understood. Thus, elucidating this mechanism would pave the way for establishing novel strategies in managing bacterial infection in aquaculture.

We recently proposed to reprogram metabolomics to boost the immune response of the host to cope with bacterial infection and clear antibiotic-resistant bacteria [10,11]. We and others have identified that exogenous glycine empowers host to clear serum-resistant bacteria, which increases host survival by 40%-60% [12]. Other metabolites including proline, leucine, glucose, serine, stearic acid, and palmitic acid were also found to increase the tilapia survival rate from 14% to 60% against infection by *Streptococcus iniae, S. agalactiae,* or *Edwardsiella tarda* [7,13–16]. Malic acid, phenylalanine, palmitic acid, L-aspartic acid increased Zebrafish survival rate from 20% to 47% against infection by *Vibrio alginolyticus* or *E. tarda* [10,17–19]. In addition, exogenous taurine, threonine, and palmitic acid increased the crucian carp survival rate from 22% to 38.6% against *E. tarda* infection [20]. These results together suggested that metabolites are potential regulators of immune response. Thus, temperature may influence immune response via metabolism.

To investigate this possibility, we established the crucian carp-*Aeromonas sobria* infection model and examine the metabolism changes and immune response in host grown at different temperatures. Crucian carp, *Carassius auratus*, is one of the most extensively cultured freshwater fish species throughout the world, reaching 2.2 million tons in the year 2010, and has great economic value [21]. More importantly, crucian carp exhibits a striking capacity to cope with the low level of oxygen and a wide range of ambient temperatures. Thus, the capability of crucian carp to adapt to versatile environments suggests that it is an ideal model to investigate the impacts of environmental factors on its immune responses to pathogens [22]. Indeed, crucian carp has proved to be a good model in studying genome evolution and physiological adaptation [23].

Aeromonas spp. causes hemorrhagic septicemia, ulcerations of the skin, and gastrointestinal tract infections in various fish hosts including crucian carp [24,25]. The infection by Aeromonas spp. often occurs when culture conditions to fish, such as water quality, temperature, were changed dramatically [6]. Aeromonas spp. not only cause massive death in fish but also in other aquaculture animals [26,27]. *A. sobria* is a representative pathogen of Aeromonas spp that calls for emergency attention [28].

In this study, we adopted the crucian Carp-*A. sobria* infection model and performed a functional metabolomic analysis. We found that crucian carp had different susceptibilities to *A. sobria* infection at different temperatures and associated with distinct metabolomic profiling of the fish. Importantly, maltose was identified as one of the most crucial biomarkers whose abundance was highly suppressed in fish cultured at a higher temperature. Fish injected by exogenous maltose demonstrated increased resistance to *A. sobria* infection at a higher temperature, likely via selectively regulating the expression of immune genes.

## Materials and methods

### Ethics statement

This study was conducted following the recommendations in the Guide for the Care and Use of Laboratory Animals of the National Institutes of Health and maintained according to the standard protocols. All experiments were approved by the Institutional Animal Care and Use Committee of Sun Yat-sen University (Animal welfare Assurance Number: 16).

### Fish maintenance

Crucian carp with an average body weight of 2 ± 0.2 g were obtained from a local crucian carp breeding corporation (Guangzhou, P.R. China), and were free of *A. sobrial* infection through microbiological detection. They were maintained in 50 L open-circuit water tanks equipped with Closed Recirculating Aquaculture Systems. Crucian carp were fed twice daily with commercial fish feed (38% crude protein, 6% crude fat, and 16% crude ash related to the wet matter, 7% crude fiber, and 8% moisture, based on nutritional research council (NRC) recommendations, at a ratio of 3% of body weight per day) on a 12 h/12 h rhythm of light and darkness photoperiod always. The tank was cleaned once a day by siphoning up the food debris and feces. To investigate the fish susceptibility to *A. sobrial* at different temperatures, crucian carp were acclimated to either 18°C or 33°C with dissolved oxygen at 7 ± 0.5 mg/L for 2 weeks before the experiments.

### Bacterial infection

For *A. sobrial* infection, a single colony of *A. sobrial* was picked up from the agar plate and grown in LB medium overnight at 30°C shaking at 200 rpm. The overnight culture was then re-inoculated to fresh LB medium at a ratio of 1:100 and grew with shaking at 200 rpm until OD600 reached 1.0. Cells were pelleted by centrifugation, washed with 0.85% saline solution three times and suspended in saline solution.

For bacterial infection, crucian carp were individually challenged with 10 μl of 1 × 10^6^ CFU bacterial suspension or saline only via intraperitoneal injection (n = 30 for each treatment). Fish mortality was examined twice daily for 15 days to monitor accumulative death.

### Sample preparation for gas chromatography-mass spectrometry (GC-MS) analysis

The sample preparation was carried out as previously described [10]. Crucian carp were euthanized in ice slush for at least 10 min until the cessation of the gill movement. Fish were then left in the ice water for a total of 20 min to ensure death by hypoxia. The individual spleen surgically isolated were quickly rinsed with cold saline buffer to remove blood, followed by immersing in cold methanol at 800 μl/100 mg. The whole spleen was homogenized for 8 mins in methanol, followed by sonication for 5 min at a 10 W power setting. A total of 60 crucian carp accommodated at 18°C or 33°C were used in this experiment (n = 30 for each group). Spleens from three crucian carp were pooled as one biological sample. Samples were then centrifuged to remove unresolved matter at 12,000 × g, 4°C for 10 mins. The supernatant was collected and 10 μl 0.1 mg/mL ribitol (Sigma) was added as an internal standard. Afterward, the aqueous sample was concentrated in a rotary vacuum centrifuge device FreeZone® (LABCONCO) for 4 h, and the resultant dried extracts were used for GC-MS analysis.

### GC-MS analysis

The GC-MS analysis was carried out following the two-stage techniques with minor modifications [10]. In brief, samples were derivatized in 40 μl of 20 mg/mL methoxyamine hydrochloride (Sigma Aldrich) in pyridine to protect carbonyl moieties through methoximation for 90 min at 37°C, followed by adding 80 μl of N-methyl-N-trimethylsilyl tri fluoroacetamide (MSTFA) (Sigma Aldrich) for derivatization of acidic protons at 37°C for another 30 min. The derivatized sample of 1 μl was injected into a 30 m × 250 μm i.d. × 0.25 μm DBS-MS column using splitless injection, and analysis was carried out in Agilent 7890A GC equipped with an Agilent 5975 C VL MSD detector, Agilent Technologies. The initial temperature of the GC oven was held at 85°C for 5 min followed by an increase to 270°C at a rate of 15°C min^−1^, and then held for 5 min. Helium was used as the carrier gas and the flow rate was kept constantly at 1 mL min^−1^. The MS was operated in a range of 50–600 m/z. For each sample, two technical replicates were prepared to confirm the reproducibility of the reported procedures.

### Exogenous addition of maltose and bacterial challenge

Crucian carp (n = 120) were randomly divided into two groups, acclimatized for 7 days at 33°C. Thirty individuals were included in each group. As the fish were in similar weight, each fish was injected with 10 μl 0.85% saline solution as control group or with 150 μg, 300 μg or 600 μg maltose as the experimental groups. The fish were injected once daily for 3 days. After the treatment, fish were challenged by intraperitoneal inoculation of 1 × 10^6^CFU/fish *A. sobrial*. Crucian carp were observed for 15 days for accumulative death.

### Measurement of enzymatic activities

Spleens from the crucian carp were broken by sonication for 5 min at a 10 W power setting in cold 1× PBS, followed by centrifugation at 12000 × g, 4°C for 10 min. The supernatant containing 100 μg total proteins were applied for pyruvate dehydrogenase and succinate dehydrogenase, 250 μg total proteins for α-ketoglutarate dehydrogenase and malic dehydrogenase, then they were transferred to the PHD and KGDH reaction mix (0.5 mM MTT, 1 mM MgCl2,6.5 mM PMS, 0.2 mM TPP, 50 mM PBS, and 2 mM sodium pyruvate for PDH or 2 mM sodium a-Ketoglutaric for KGDH) or SDH reaction mix (0.5 mM MTT,13 mM PMS, 5 mM succinate, 50 mM PBS) to a final volume of 200 μl in96-well plate. Subsequently, the plate was incubated at 37°C for 15 min, and then each plate was measured at 570 nm for colorimetric reading.

### RNA isolation and qRT-PCR

Total RNA of spleen was isolated with Trizol (Invitrogen, USA). The isolated RNA was then quantified by detecting the intensity of fluorescence. Reverse transcription-PCR was carried out on a Primer-Script^TM^ RT reagent kit with a gDNA eraser (Takara, Japan) with 1 μg of total RNA according to manufacturer’s instructions. The experiment was performed in four biologicals obtained.

qRT-PCR was performed as described previously [10]. Primers for each gene were listed in Table 1. Each primer pair was specific, the relative expression of each gene was determined by the comparative threshold cycle method (2^−ΔΔCT^ method), and the resulted Ct values were summarized in Supplementary Figure 1 [29].

**Table 1.** Primers used for QRT-PCR analysis.

Gene	Primer	Sequence (5’-3’)
*actin*	Forward	gggatgggacagaaggacag
	Reverse	acgcagctcgttgtagaagg
*gapdh*	Forward	tgacccctccagtatgacca
	Reverse	gagggcctcctcaataccaa
*tubulin*	Forward	ctgctgggaactctattgtc
	Reverse	ctccaggtctacaaacacag
*il1b1*	Forward	atgcgctgctcaacttcat
	Reverse	ctggcccttattttgttgag
*il1b2*	Forward	caaagcgatcctcttcattt
	Reverse	attcgggtcatcagttttaa
*il11*	Forward	ttcgagtggctgaacagaac
	Reverse	aggcccagtcacagaagagc
*tnfα1*	Forward	tcacgctcaacaagtctcag
	Reverse	tggtcctttctccagtaaag
*tnfα2*	Forward	ccgctgtctgcttcacatt
	Reverse	ggccttggaagtgacattt
*tlr2*	Forward	cttagatgggctcactcatc
	Reverse	gggtgggagacatctttaag
*tlr3*	Forward	tagatgccagctacaactcttt
	Reverse	ggctccccaattaacttcag
*tlr9*	Forward	gccaacccatgttatcagtc
	Reverse	ggtgtcgcagatttttaaga
*nfkbiab*	Forward	cagtttggcgcagacatt
	Reverse	gcgcctttgctgattagaag
*ifnγ1-1*	Forward	ctacgggtcctgaaagactt
	Reverse	gcctgggaagtagttttctc
*ifnγ1-2*	Forward	tctggggagtatgcttgttga
	Reverse	gcctgggaagtagttttcttg
*c3*	Forward	tggggatggatctgaaaca
	Reverse	tgcccatgatgaggtacga
*lyz*	Forward	tgtgtctgatgtggctgtgc
	Reverse	tgcacacatagttgccaagtga

## Results

### Crucian carp cultured at 33°C were more susceptible to *A. sobrial*

**Figure 1.**
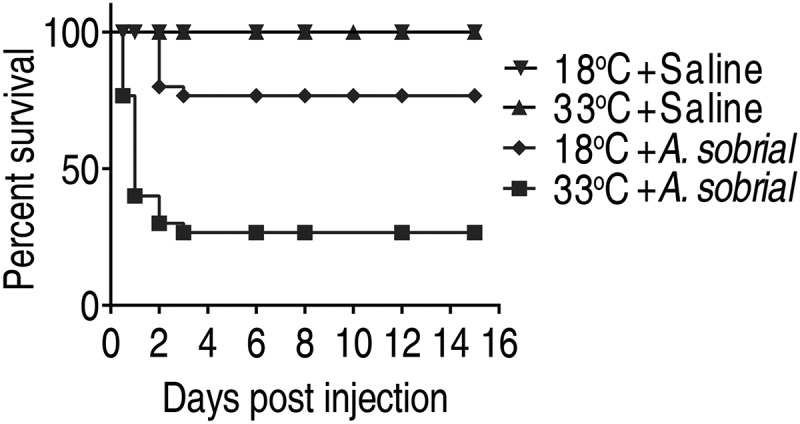
Percent survival of crucian carp to *A.sobrial* infection at 18°C and 33°C. Crucian carp were acclimated at 18°C or 33°C for 7 days and then were challenged with saline or *A.sobrial* (1 × 10^6^CFU/dose; n = 30 for each treatment). The percent survival was monitored for 15 days.

To investigate how temperature influences the susceptibility of crucian carp to *A. sobrial* infection, crucian carp, acclimated at 18°C or 33°C for 7 days, were infected with *A. sobrial*. Crucian carp started to die from 12 h and stabilized after day 3 (Figure 1). Crucian carp grown at 33°C was more susceptible to *A. sobrial* infection than those grown at 18°C. The percent survival is 27% at 33°C, and 76% at 18°C (Figure 1). Crucian carp that were injected with 0.85% saline solution served as the negative control, which did not die in the entire period of the experiment.

### Differential metabolomes of crucian carps at 33°C and 18°C

To explore the metabolic mechanism of why crucian carp survived better to *A. sobrial* infection at 18°C, we profiled the metabolome of crucian carp that were grown at these two different temperatures. By gas chromatography-mass spectrometry (GC-MS)-based metabolomic approach, the metabolome of spleens from the two groups was compared. Ten biological replicates and two technical replicas of each group were performed, yielding 60 data sets. We obtained a total of 236 peaks for each sample (Supplementary Figure 2A). After removing the internal standard, ribitol, deleting the solvent peaks, and merging the same compounds, 58 metabolites were identified. Crucian carp grown at 18°C and 33°C demonstrated distinct metabolome, and a heat-map was generated to show the relative abundance of each metabolite in Supplementary Figure 2D. The Pearson correlation coefficient between two technical replicates varied 0.989 and 0.998 (Supplementary Figure 2B), which suggests that the reproducibility of GC-MS was sufficient to ensure data quality in global metabolic profiling application. The identified metabolites were categorized as carbohydrates (26%), lipids (29%), amino acids (24%), nucleotides (9%), and others (12%) (Supplementary Figure 2C).

**Figure 2.**
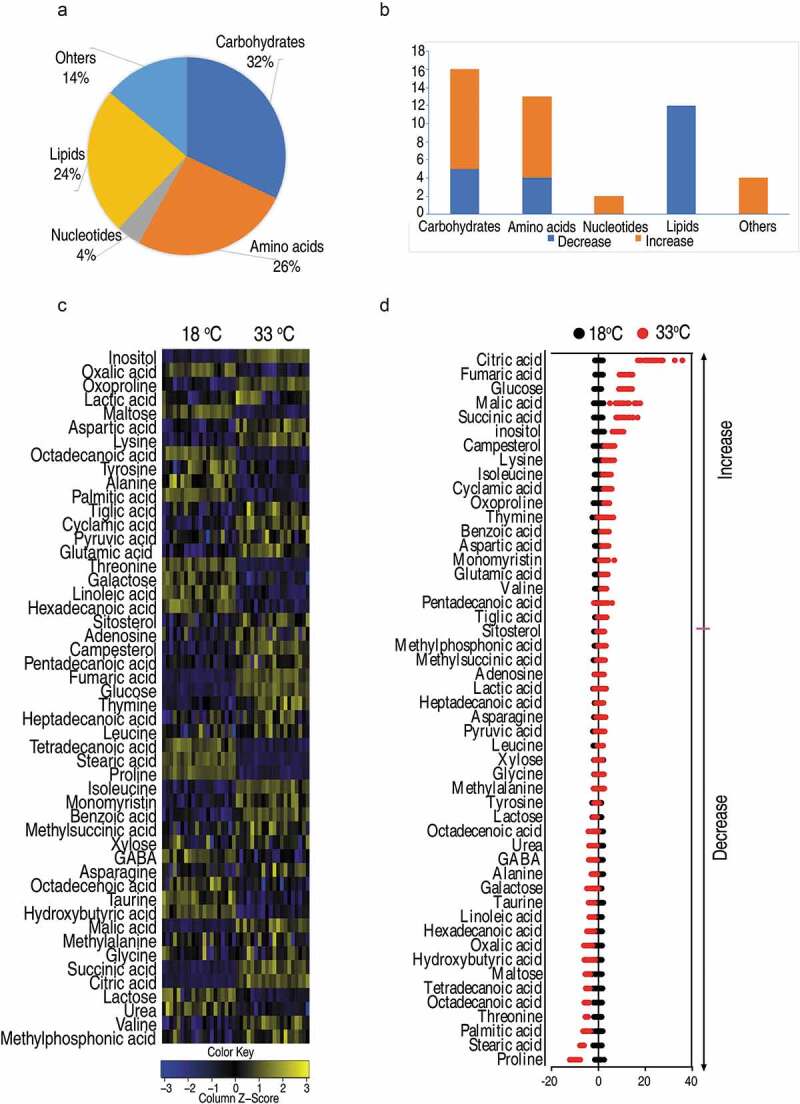
*Crucian carp* had different metabolomics profiling when cultured at different temperatures.

By comparing the metabolomes of 18°C and 33°C, 50 metabolites had significant differential abundance (Figure 2(c)). The Z-score varied between −10 and 24 in the 33°C compared with the 18°C. Specifically, the abundance of 31 metabolites increased and of 19 metabolites decreased in the 33°C samples (Figure 2(d)). These metabolites belong to carbohydrates (32%), amino acids (26%), nucleotides (4%), lipids (24%), and others (14%) (Figure 2(a)). The abundance of all the identified lipids was decreased in the 33°C group while all the identified nucleotides were increased in 33°C (Figure 2(b)), implying crucian carp adjust their metabolism when cultured at different temperatures that may influence their susceptibility to *A. sobrial* infection.

(a) Categories of the differential metabolites. (b) The number of differential abundance of metabolites in each category. Blue or orange indicates a decrease or increase in the abundance of metabolites, respectively. (c) Heat map of unsupervised hierarchical clustering of differential metabolites (row). Yellow and blue indicate the increase and decrease of the metabolites scaled to mean and standard deviation of row metabolite level, respectively (see color scale). (d) Z scores (standard deviation from average) of 33°C-group to 18°C-group which are corresponding to the data shown in (c). Each point represents one technical repeat of metabolite.

### Differential metabolic pathways of crucian carps at 33°C compared to that at 18°C

**Figure 3.**
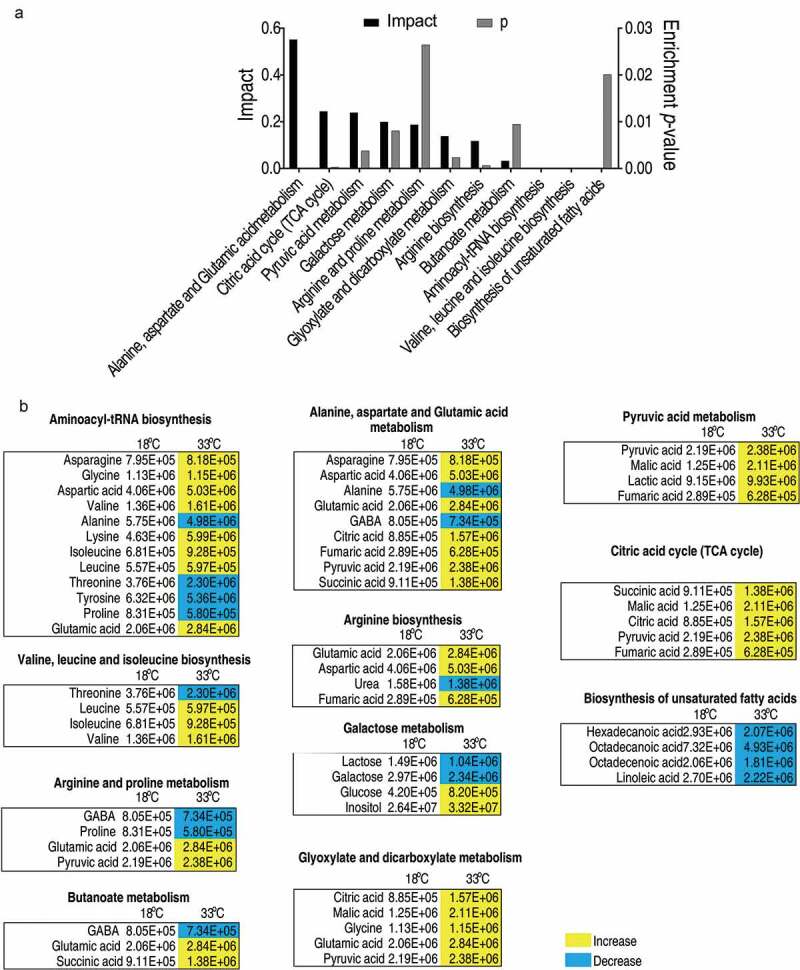
Pathway analysis of differential metabolites.

To explore the metabolic pathways that distinguish crucian carp grown at 18°C and 33°C, ingenuity network analysis was performed to analyze the metabolites of differential abundance. Eleven metabolic pathways were enriched including alanine, aspartate, and glutamic acid metabolism, citric acid cycle (TCA cycle), pyruvic acid metabolism, galactose metabolism, arginine and proline metabolism, glyoxylate and dicarboxylate metabolism, arginine biosynthesis, butanoate metabolism, aminoacyl-tRNA biosynthesis, valine, leucine, and isoleucine biosynthesis as well as the biosynthesis of unsaturated fatty acids (Figure 3(a)). The first three pathways were the most enriched.

(a) Pathway enrichment analysis of differential metabolites. Significantly enriched pathways are selected to plot (*p* value<0.05), and their impact was indicated. (b) The relative abundance of metabolites of each pathway listed in (a). Metabolites highlighted with yellow and blue indicate the increased and decreased abundance, respectively.

**Figure 4.**
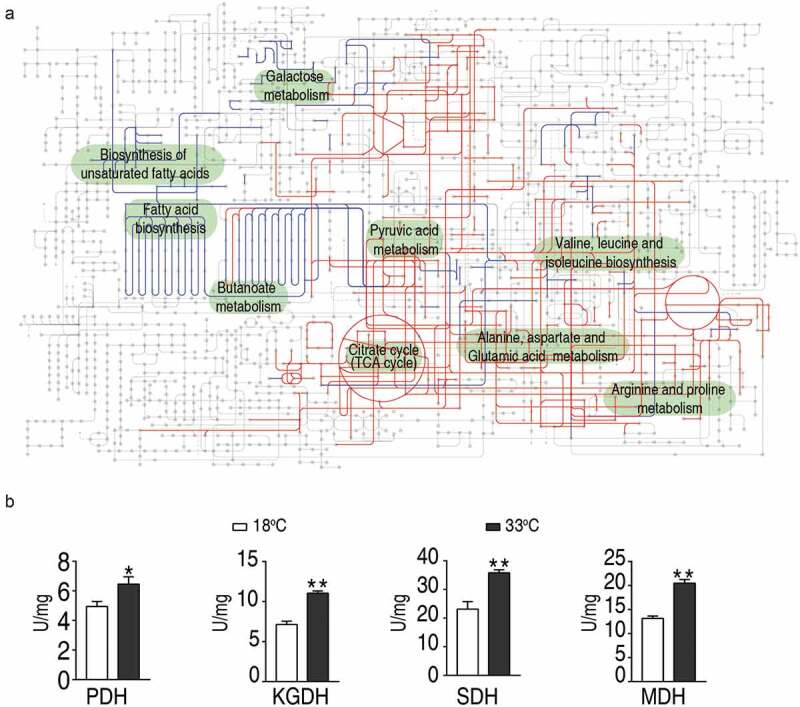
Metabolic network of the metabolites with differential abundance, and measurement of the activities of enzymes of the TCA cycle.

To gain a global view of the adjusted metabolism, all metabolites demonstrating differential abundance were integrated to generate the iPath as shown in Figure 4(a). All the enriched pathways can be visualized, which were all decreased except that for the biosynthesis of fatty acids. Among all the pathways, the TCA cycle was of particular interest, as the abundance of all the identified metabolites was increased in the 33°C (Figure 3(a and b)). To confirm the TCA cycle was modulated, the activities of four enzymes in the TCA cycle, i.e. succinate dehydrogenase (SDH), α-ketoglutarate dehydrogenase (α-KGDH) and malic dehydrogenase (MDH), and pyruvate dehydrogenases (PDH) that connects the glycolysis and the TCA cycle, were quantified. Interestingly, all of the four enzymes demonstrated elevated enzymic activity at 33°C than those at 18 ^o^C, with an increase of 30.9% for PDH, 54.9% for KGDH, 55.0% for SDH, and 55.7% for MDH (Figure 4(b)). Thus, the elevated activity of TCA cycle is an outstanding metabolic feature of crucian carp when grown at high temperatures.

(a) Integrated metabolic network in relation to differential metabolites by iPath. The red and blue are depicted the increased and decreased metabolites in 33°C-group, respectively (b) The enzymatic activity of PDH, α-KGDH, SDH, and MDH of spleens isolated from crucian carp grown at 33°C and 18°C. Values are means ±SEM from 6 biological replicates as analyzed by Kruskal–Wallis followed by Dunn’s multiple comparison post hoc test. * p < 0.05; ** p < 0.01

### Identification of metabolic biomarkers in crucian carp grown at different temperatures

**Figure 5.**
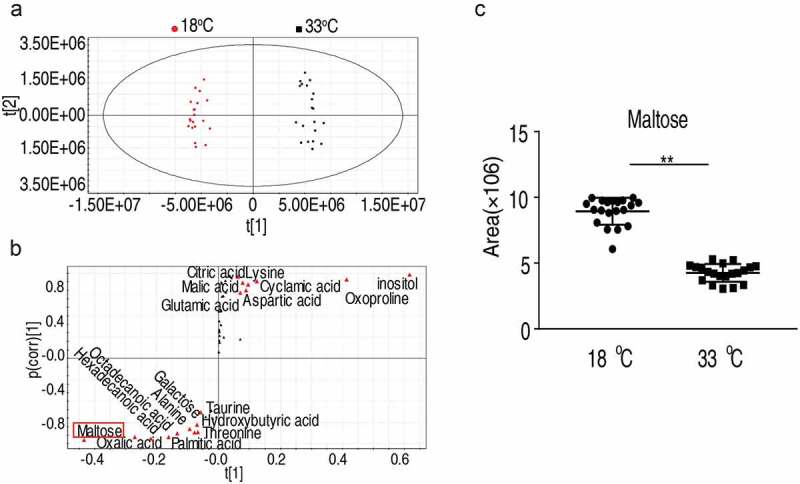
Maltose promotes fish survival against *A. sobrial* infection.

To identify the most crucial metabolites that differentiate(s) 33°C samples from the 18°C samples, orthogonal partial least square discriminant analysis (OPLS-DA) was conducted for multivariate analysis. The two groups were individually clustered together, demonstrating that the sample from fish grown at 33°C and 18°C has a distinct metabolic signature (Figure 5(a)). Additionally, there were no significant outliers were detected, ensuring the quality of the sample for further analysis (Figure 4(a)). Discriminating variables were shown as S-plot in Figure 4(b), and the cutoff values were set as greater or equal to 0.05 and 0.5 for the absolute value of covariance p and correlation p(corr), respectively. The crucial biomarkers screened by component p [1] were shown with a red triangle in Figure 5(b). A total of 18 crucial biomarkers were identified and displayed as a scatter plot (Supplementary Figure 2). Maltose was one of the most outstanding metabolites, whose abundance was largely suppressed in the 33°C group (Figure 5(c)). These data suggest that maltose likely is a crucial metabolite that determines crucian carp survival upon *A. sobrial* infection.

(a) The PCA analysis of metabolomic profiling of sample from fish grown at 33°C and 18°C. Each dot represents the technique replicates in the plot. (b) S-plot, generated by OPLS-DA, to identify differential metabolites of intragroup as from t [1] in (a). Each triangle represents individual metabolite, where potential biomarkers are highlighted with red, which is greater or equal to 0.05 and 0.5 for the absolute value of covariance p and correlation p (corr), respectively. (c) Maltose is a crucial biomarker that distinguishes the 33°C and 18°C as shown with dot-blot.

### Maltose increases resistance of crucian carp to *A. sobrial i*nfection at 33°C

**Figure 6.**
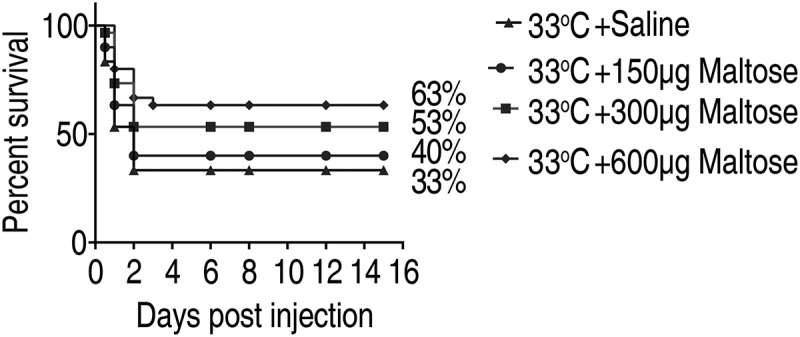
Maltose promotes fish survival against *A.sobrial* infection.

To investigate the potential role of maltose on crucian carp survival against *A. sobrial* infection, crucian carp grown at 33°C were intraperitoneally injected with three doses of maltose (150 μg, 300 μg or 600 μg per fish). Then, crucian carp were challenged with *A. sobrial*. The percent survival of crucian carp to *A. sobrial* infection was increased from 33% to 63% in a dose-dependent manner (Figure 6). Thus, maltose is a metabolite that could promote crucian carp survival at high temperature.

Percent survival of crucian carps in the presence of maltose. Crucian carp were treated with saline or different doses of maltose at 33°C for 3 days, followed by bacterial challenge through intraperitoneal injection (1 × 10^6^ CFU). The accumulative fish death was monitored for a total of 15 days post-infection (n = 30 per group).

### Maltose does not affect the TCA cycle

**Figure 7.**
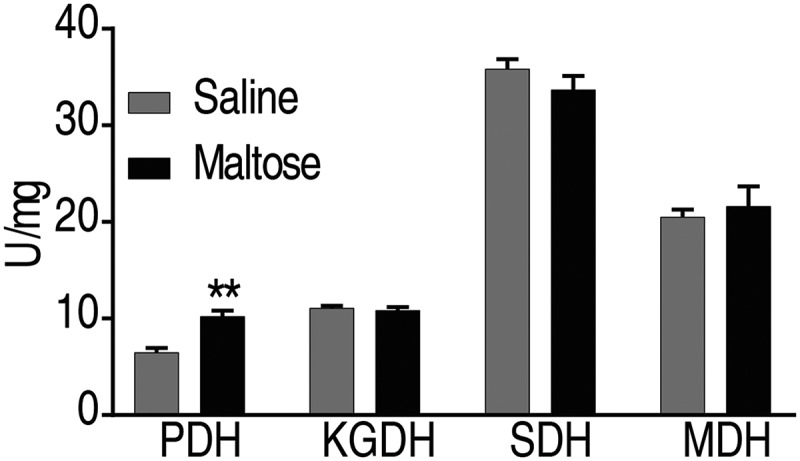
Maltose does not promote the activity of TCA cycle.

Maltose is a disaccharide that can be broken into monosaccharide of glucose. Glucose can be further metabolized via the glycolytic pathway for the generation of Acetyl-CoA, which fuels the TCA cycle or is the source for fatty acid biosynthesis. We observed that maltose only increased the activity of PDH, but not SDH, α-KGDH, and MDH (Figure 7). These data suggest that maltose promotes fish survival not via elevating the activities of the TCA cycle.

The enzymatic activity of PDH, α-KGDH, SDH, and MDH of spleens isolated from crucian carp after administration of maltose (n = 6; 600 μg) at 33°C. Values are means ±SEM from 6 biological replicates as analyzed by Kruskal–Wallis followed by Dunn’s multiple comparison post hoc test. * p < 0.05; ** p < 0.01

### Crucial biomarkers modulate the innate immune response of crucian carps at 33°C

**Figure 8.**
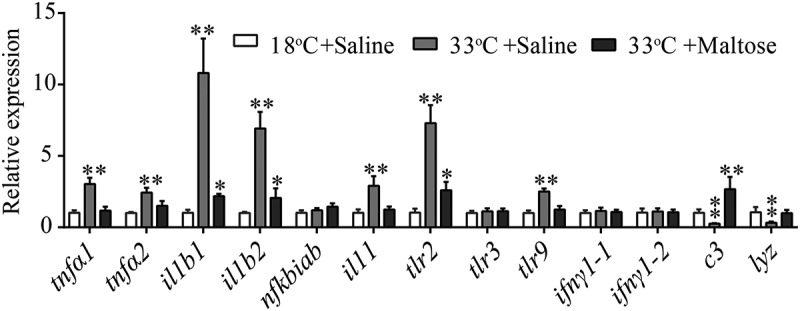
Maltose modulates innate immune response at 33°C.

To investigate whether maltose could potentiate immune response that may increase crucian carp survival, the expression of immune genes, *tnfα1, tnfα2, ilb1, ilb2, lyz, ifnγ1-1, infγ1-2, nfkbiab, il11, c3, tlr9, tlr2, and tlr3* were quantified. Crucian carp was injected 500 μg maltose or the same volume of saline as the negative control and then challenged with 1 × 10^4^ CFU/dose of *A. sobrial*. Spleens were removed for gene expression analysis. Interestingly, the expression level of cytokine, including *tnfα1, tnfα2, ilb1, ilb2, il11*, and *tlr2*, was higher in 33°C compared to those from 18°C. Maltose, however, downregulates the cytokine expressions at 33°C to a level like those at 18°C. Interestingly, the expressions of lysosome (*lyz*) and complement component *c3* were significantly lower in fish grown 33°C and got boosted when treated with maltose (Figure 8). These data suggest that maltose might selectively modulate the expression of genes to enhance crucian carp to fight against *A. sobrial* infection.

qRT-PCR for cytokine genes of *crucian carp* treated with saline or maltose (600 μg) for 3 days following with *A.sobrial* challenge through intraperitoneal injection (1 × 10^4^ CFU). Values are means ±SEM from six biological replicates. * p < 0.05; ** p < 0.01.

## Discussion

Environmental factors like temperature, acidity, salinity, and oxygen capacity are profound factors influencing the spread of transmissible disease in aquaculture [30]. The environmental temperature changes, for example, have important impacts on the physiology of animals and their immunity, which ultimately affects their resistance to pathogens [31]. Although strategies including antimicrobial compounds, vaccination, probiotics, and immunomodulatory are attractive targets to control a bacterial infection, they all face up issues, including the emergence of drug-resistant bacteria, environmental contamination, time- and labor-consuming, species-specific or excessive immune response, that limit their wide usages [32–34]. Therefore, the development of the eco-friendly approach, especially under unfavorable cultural conditions like high environmental temperature, is urgently needed.

In the present study, we propose that exogenous metabolites could reinforce fish’s immunity to fight against bacterial infection when grown at a high temperature, which we termed reprogramming metabolomics [35]. This strategy is based on the comparison of the metabolomes in fish grown at different environmental temperature condition, from which we identify the crucial biomarker of maltose and apply this metabolite to reprogrammed the host metabolome. As compared to other strategies mentioned above, this approach relies on the metabolites that commonly presents in the host; thus, it is nontoxic, non-immunogenic, and eco-friendly. We have previously shown that this approach not only increased the resistance of hosts to bacterial infection but also kill multidrug-resistant bacteria in the combination of antibiotics in the model of fish and mouse [12,16,36]. In this study, exogenous maltose increased the crucian carp survival rate by 30%, thus representing an alternative strategy for current therapy for bacterial infection.

Maltose is a known disaccharide that is generated during the intestinal digestion or glycolysis of glycogen and starch. But the function of maltose in immunity is less documented. Here we found that maltose could regulate the expression of immune gene expression. Of special interest, maltose downregulates the cytokine production including *tnfα1, tnfα2, il1b1, il1b2, il11, tlr2, and tlr9*. Importantly, it also increases the production of the humoral innate immune mediators including lysozyme and complement component *c3*. The upregulation of lysozyme is consistent with previously reported that the supplementation of maltose in fish food increased the serum level of lysozyme [37]. This differential regulation of immune response by maltose indicates that maltose likely fine tune the expression of cytokines to prevent excessive immune response as *tnfα, ilb1b, and il1b2*, which contribute significantly to cytokine storm during bacterial infection and result in organ failure [38–40]. We thus speculate that some of the fish died due to not the high virulence of the bacteria but the excessive immune response. These excessive immune responses can be counteracted by the provided maltose. In addition, maltose is also an ingredient of human milk. Other disaccharides in human milk, such as trehalose, lactose, and galactose, substantially increased the production of cathelicidin antimicrobial peptide LL-37 in HT-29 cell line, which contributes to intestinal homeostasis [41].

It is interesting to observe that the TCA cycle is upregulated when fish were grown at 33°C compared to 18°C as demonstrated by both of the abundances of TCA cycle metabolites (citric acid, malic acid, succinic acid, pyruvic acid, and fumaric acid) and the enzymatic activity (KGDH, SDH, and MDH). This is consistent with the notion that elevated temperature increases metabolism, respiration, and oxygen. However, the increased TCA cycle activity makes fish more susceptible to *A. sobrial* infection.

Although the mechanism is required for further investigation, we speculate that metabolic flux flow to the biosynthesis of fatty acid may be augmented fish survival to *A. sobrial* infection. In this study, we observed that maltose increased the glycolysis as demonstrated by increased PDH activity (Figure 7), implying maltose is broken down to glucose and flux to the glycolysis. However, the activity of enzymes of the TCA cycle was not upregulated, indicating that pyruvate, the product of PDH, did not enter the TCA cycle, instead, possibly into the biosynthesis of fatty acids, whose abundance were all downregulated in fish cultured at 33°C. Therefore, it is possible that maltose exerts anti-infection ability via fatty acids but not the TCA cycle. Actually, fatty acids are stimulators to the immune response [18,42,43]. Fatty acids may exert their immune-stimulatory functions through diffusion of the membrane of T-cells in a passive process or active transport through cellular receptors or bind to cellular receptors like toll-like receptors [44]. By this way, fatty acids are active regulators of phagocytosis, NLRP3 inflammasome activation, macrophage frequency, M-2 phenotype shift, and cytokine production [45]. Consistently, we have previously shown that palmitic acid and stearic acid protect tilapia against *E. tarda* infection [46].

With the potential of maltose in increasing innate immune response, we expect it can be used as an adjuvant during vaccination. C3, for example, is a component of the complement system that bridges the innate immunity and adaptive immunity. Growing evidences showed that B cell expressed C3 receptor on their cell surfaces and their activations trigger B cell differentiation to produce antibodies and form long-term memory [47,48]. Exogenous maltose is possible to augment antibody production during vaccination.

In conclusion, we have demonstrated that crucian carp grown at 33°C have increased susceptibility to *A. sobrial* infection, which is associated with the changed metabolomic profiling. The identified crucial biomarker, maltose, could enable crucian carp to cope with *A. sobrial* infection at such temperature. This efficacy is likely achieved by enhancing specific immune gene expression. Therefore, our study represents a novel approach to manage *A. sobrial* infection of crucian carp in unfavored growth conditions.

## Supplementary Material

Supplemental Material
