# Supplementary material for: Maltose promotes crucian carp survival against Aeromonas sobrial infection at high temperature
Source: Virulence. 2020 Jul 22;11(1):877–88. doi: 10.1080/21505594.2020.1787604 (PMC7549911; doi:10.1080/21505594.2020.1787604)
Supplement: Supplemental Material [file KVIR_A_1787604_SM3901.zip › Supplementary Figure Legends.docx]

**Supplementary Figure Legends**

**Figure 1. Ct values of each gene.** The Ct value of each quantified gene were displayed as histogram.

**Figure. 2. Metabolomic analysis of crucian carp grown at 18^o^C and 33 ^o^C. (**A) Chromatograms of GC-MS. (B) Correlation coefficient of different replicates. (C) Functional categorization of identified metabolites (D)Heat-map of identified metabolites.

**Figure 3. Scatter plot of crucial biomarkers.** The abundance of each crucial metabolite at 18 ^o^C and 33 ^o^C were displayed as scatter plot.
