# Supplementary material for: Maltose promotes crucian carp survival against Aeromonas sobrial infection at high temperature
Source: Virulence. 2020 Jul 22;11(1):877–88. doi: 10.1080/21505594.2020.1787604 (PMC7549911; doi:10.1080/21505594.2020.1787604)
Supplement: Supplemental Material [file KVIR_A_1787604_SM3901.zip › Supplementary Table.docx]

| Table 1 Primers used for QRT-PCR analysis. | | |
| --- | --- | --- |
| Gene | Primer | Sequence(5'-3'） |
| *actin* | Forward | gggatgggacagaaggacag |
|  | Reverse | acgcagctcgttgtagaagg |
| *gapdh* | Forward | tgacccctccagtatgacca |
|  | Reverse | gagggcctcctcaataccaa |
| *tubulin* | Forward | ctgctgggaactctattgtc |
|  | Reverse | ctccaggtctacaaacacag |
| *il1b1* | Forward | atgcgctgctcaacttcat |
|  | Reverse | ctggcccttattttgttgag |
| *il1b2* | Forward | caaagcgatcctcttcattt |
|  | Reverse | attcgggtcatcagttttaa |
| *il11* | Forward | ttcgagtggctgaacagaac |
|  | Reverse | aggcccagtcacagaagagc |
| *tnfα1* | Forward | tcacgctcaacaagtctcag |
|  | Reverse | tggtcctttctccagtaaag |
| *tnfα2* | Forward | ccgctgtctgcttcacatt |
|  | Reverse | ggccttggaagtgacattt |
| *tlr2* | Forward | cttagatgggctcactcatc |
|  | Reverse | gggtgggagacatctttaag |
| *tlr3* | Forward | tagatgccagctacaactcttt |
|  | Reverse | ggctccccaattaacttcag |
| *tlr9* | Forward | gccaacccatgttatcagtc |
|  | Reverse | ggtgtcgcagatttttaaga |
| *nfkbiab* | Forward | cagtttggcgcagacatt |
|  | Reverse | gcgcctttgctgattagaag |
| *ifnγ1-1* | Forward | ctacgggtcctgaaagactt |
|  | Reverse | gcctgggaagtagttttctc |
| *ifnγ1-2* | Forward | tctggggagtatgcttgttga |
|  | Reverse | gcctgggaagtagttttcttg |
| *c3* | Forward | tggggatggatctgaaaca |
|  | Reverse | tgcccatgatgaggtacga |
| *lyz* | Forward | tgtgtctgatgtggctgtgc |
|  | Reverse | tgcacacatagttgccaagtga |
