# Supplementary figures and images for: Maltose promotes crucian carp survival against Aeromonas sobrial infection at high temperature
Source: Virulence. 2020 Jul 22;11(1):877–88. doi: 10.1080/21505594.2020.1787604 (PMC7549911; doi:10.1080/21505594.2020.1787604)

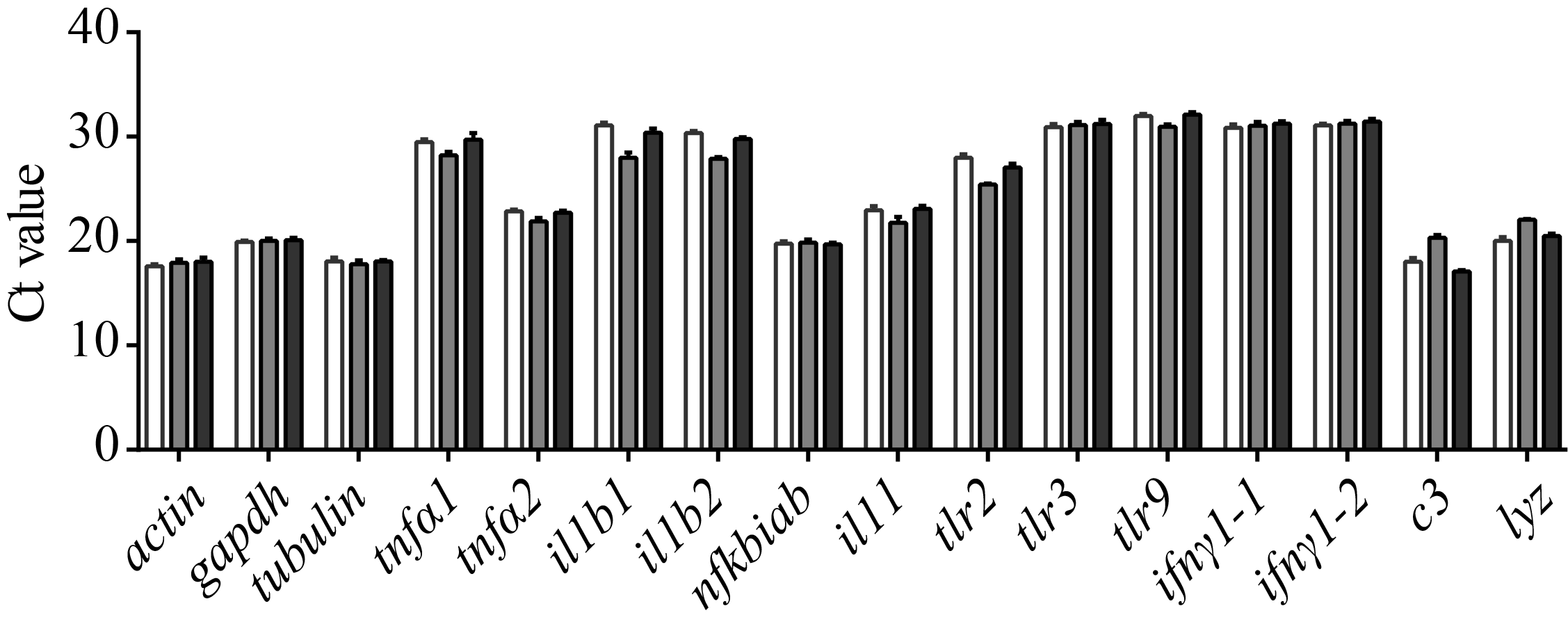

Supplement: Supplemental Material [file KVIR_A_1787604_SM3901.zip › Supplementary Figure1.png]

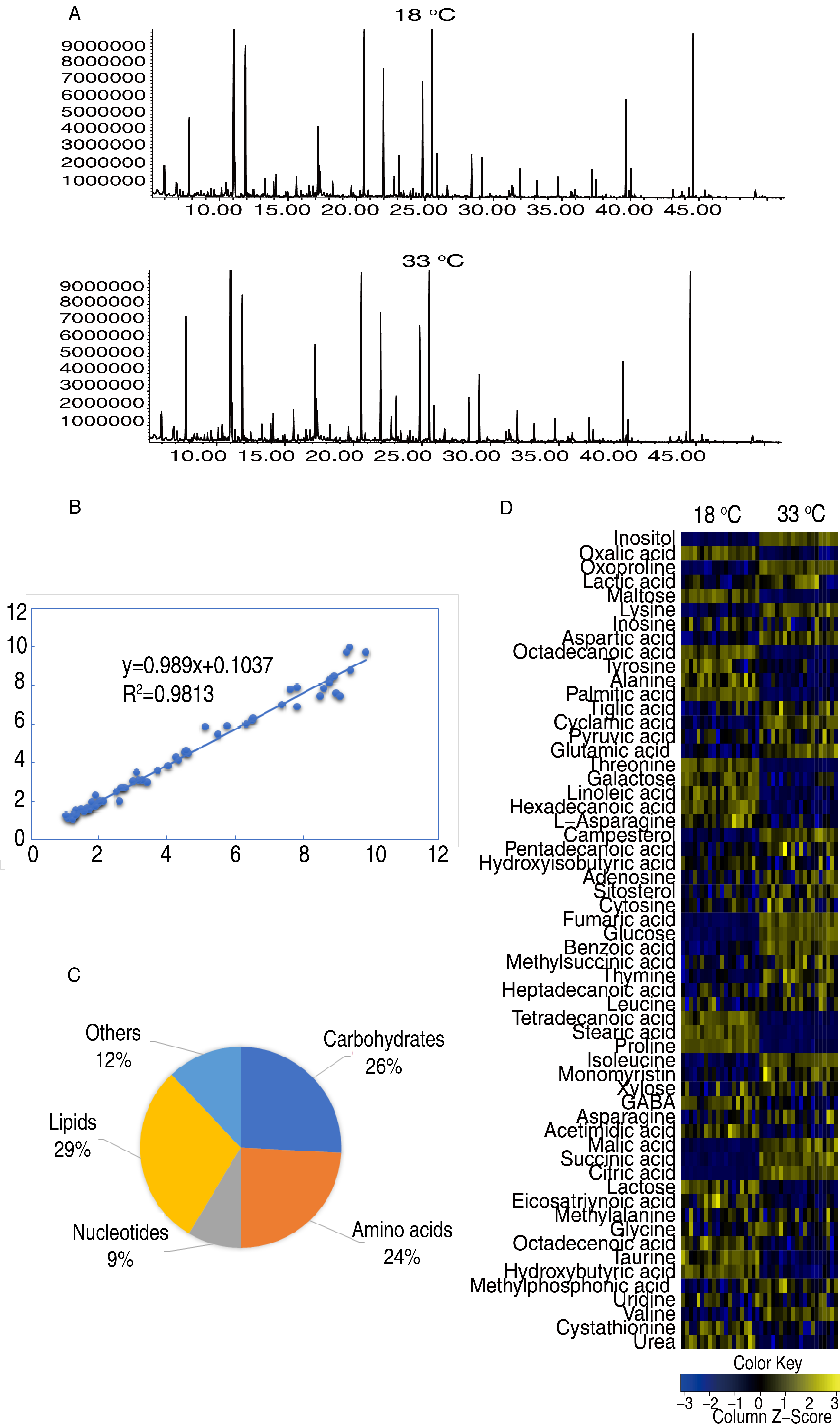

Supplement: Supplemental Material [file KVIR_A_1787604_SM3901.zip › Supplementary Figure2.png]

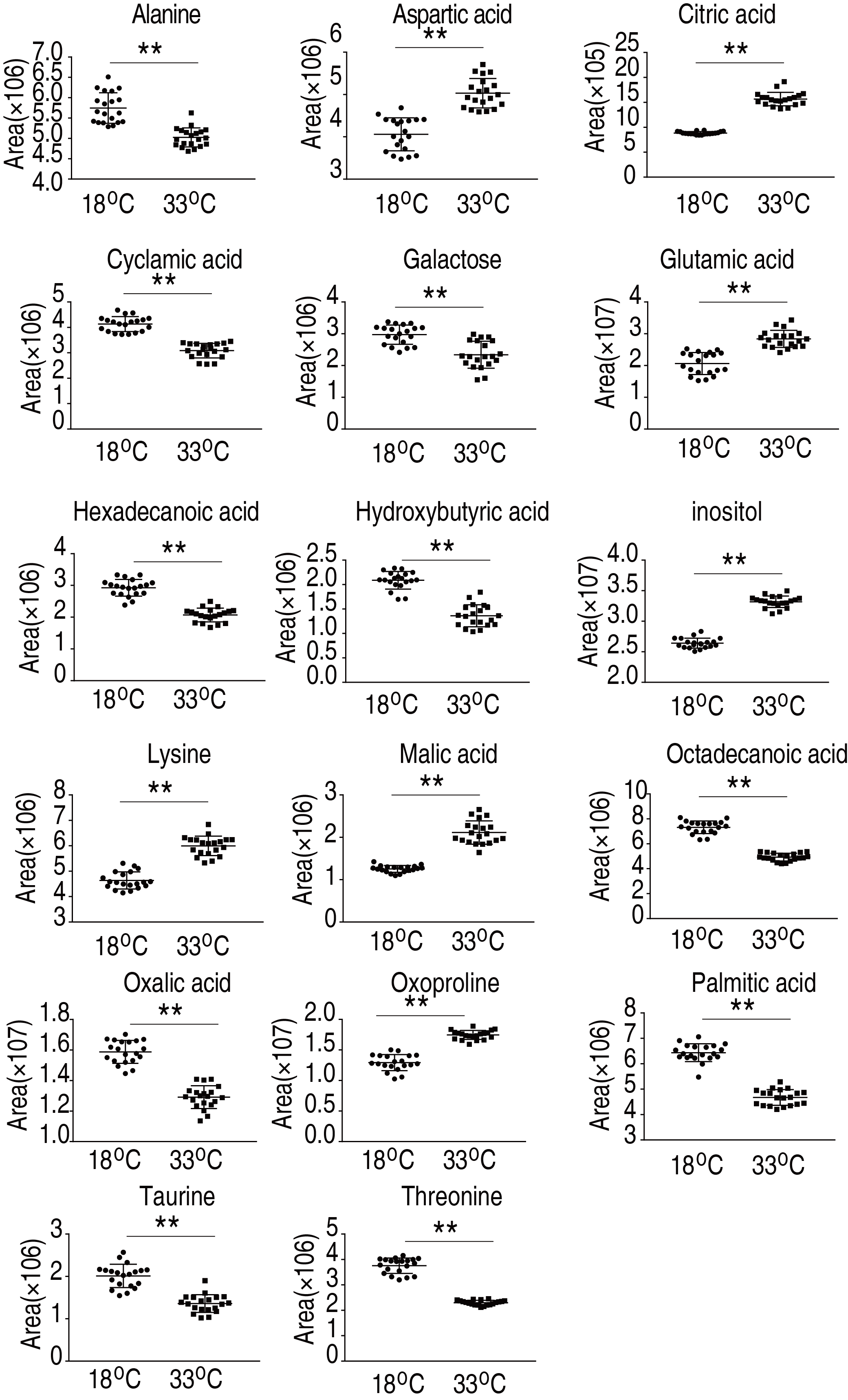

Supplement: Supplemental Material [file KVIR_A_1787604_SM3901.zip › Supplementary Figure3.png]
